# Surface Rheological Properties and Microstructures of DPPC/POPC Monolayers

**DOI:** 10.1021/acs.langmuir.5c01269

**Published:** 2025-06-20

**Authors:** Wisnu Arfian Anditya Sudjarwo, Jose Luis Toca-Herrera

**Affiliations:** Institut für Biophysik, 27270 Universität für Bodenkultur Wien (BOKU), Vienna 1190, Austria

## Abstract

In this paper, analysis of π–*A* isotherm curves, oscillatory dilatational deformation, and microstructure imaging with atomic force microscopy (AFM) has revealed complex behaviors of lipid monolayers. The π–*A* isotherm hysteresis analysis opens a new discourse on the physical properties of lipid monolayers. Isocycle curves of lipid mixtures elucidate how changes in the molecular packing affect the dynamics of the monolayer. Notably, DPPC monolayers exhibit the lowest hysteresis energy, reflecting high mechanical reversibility during compression–expansion cycles. In contrast, the introduction of POPC (i.e., as molar fractions *x*_POPC_ = 0, 0.25, 0.5, 0.75, and 1) into the lipid mixture disrupts the tight packing and increases the hysteresis. AFM images highlight the emergence of lipid domains (LE-LC phase transition and LC phase), which complement the observations from π–*A* isotherms at surface pressures of 5 and 24 mN/m. Furthermore, examination of interfacial dilatational rheology through barrier oscillation provides quantitative information about monolayer deformation. Our findings reveal that the lipid mixture, surface pressure, frequency, and amplitude sweeping influence the elastic and viscoelastic behavior of the lipid monolayer. Additionally, Lissajous plots from amplitude sweeping effectively distinguish the linear and nonlinear behavior of the monolayer.

## Graphical abstract

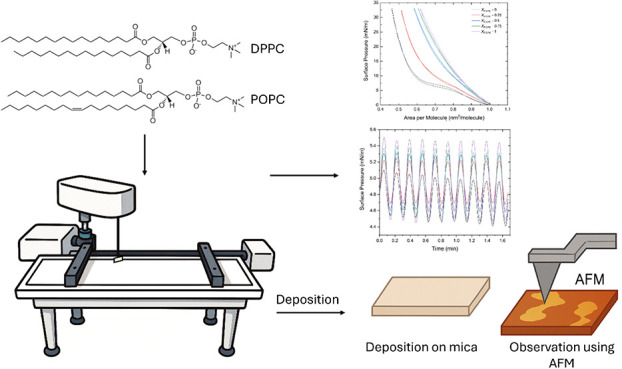

## Introduction

The stability and behavior of many complex fluids are influenced by the physical properties of the interfacial films. These films, typically composed of surfactant, reduce interfacial energy and impart viscoelasticity to the interface.^1,2^ By decreasing the interfacial tension, surfactants reduce the interfacial energy of the system. This makes the interface more stable and can help to prevent droplet coalescence. Coalescence is the merging of dispersed droplets that decreases the interfacial area. It has consequences for compressing the interfacial film or expel molecules from the surface.^3–5^ To stabilize the interfaces, surface viscoelasticity provides an essential mechanism to resist such deformations.

Surface viscoelasticity also affects the bulk rheological properties of complex fluids.^6^ The dynamic conditions reveal nonlinear rheological responses that cannot be explained by linear models typically used to describe small deformations.^7^ Understanding the nonlinear viscoelasticity of interfacial films is crucial for linking macroscopic behavior to molecular structure. Therefore, it serves deeper insights into the stabilization mechanisms of multiphase systems.^8^

Among the array of available experimental techniques, the Langmuir technique stands out as a versatile tool for investigating the interfacial behavior of lipids.^9,10^ This technique offers researchers unprecedented control over the compression and expansion of lipid monolayers at the air–water interface, facilitating a comprehensive exploration of their thermodynamic properties, e.g., phase transitions. This method involves spreading lipids at the air–water interface and compressing them using movable barriers to measure surface pressure–area (π–*A*) isotherms.^11^

In addition to π–*A* isotherms, dilatational rheology provides insight into the mechanical properties of lipid monolayers under dynamic conditions. This technique measures the monolayer’s response to oscillations in surface area, thereby providing data on the elastic and viscous properties of the film.^12,13^ These properties are critical to understanding how monolayers resist deformation and how they contribute to the stability of multiphase systems. In particular, the dilatational modulus, which describes the relationship between surface pressure and area deformation, varies significantly with composition and phase state.^14–16^ In DPPC monolayers, for instance, the elastic modulus increases sharply upon entering the LC phase due to the increased molecular packing. Meanwhile, in POPC monolayers, although the modulus increases, it does so relatively slowly with an increase in surface pressure. Other studies have revealed the relationship between the lipid composition and phase state. For instance, the elastic modulus of the DSPC/DMPC mixture increases with the increase in mole fraction of DSPC.^17^ Reports from Vranceanu et al.^18,19^ also explain that increasing surface pressure raises the elastic modulus for DPPC, DOPC, cholesterol, and even for mixtures of DPPC/cholesterol or DOPC/cholesterol.

In the present study, we extend previous findings by systematically investigating the phase behavior, thermodynamic properties, and interfacial viscoelasticity of mixed DPPC/POPC monolayers. Using the Langmuir technique, we conducted π–*A* isotherms, hysteresis analysis, and surface dilatational rheology to quantify both elastic and viscous moduli. Furthermore, we employed the Langmuir–Blodgett technique to transfer the monolayers onto mica and analyzed their microstructures using AFM imaging. This combined approach enables us to correlate lipid composition and surface pressure with molecular packing, mechanical response, and domain morphology. The DPPC/POPC system is frequently employed in model membrane studies due to its relevance to pulmonary surfactant composition, as a model to study interactions between saturated and unsaturated phospholipids.

## Experimental Section

### Materials

1,2-Dipalmitoyl-*sn*-glycero-3-phosphocholine (DPPC) and 1-palmitoyl-2-oleoyl-*sn*-glycero-3-phosphocholine (POPC) were purchased from Avanti Polar Lipids Inc. Phosphate-buffered saline (PBS) tablets, methanol, and chloroform were purchased from Carl ROTH GmbH. Milli-Q water was purified using Millipak Gold 0.22 μm filters with a final resistivity of 18.2 MΩ.cm. To obtain a 2 mM lipid solution, all phospholipids were dissolved in chloroform: methanol (4:1, v/v). To obtain 10 mM (1X) PBS at pH 7.4, the PBS tablet was dissolved in Milli-Q water and the pH was adjusted using a pH meter (Mettler-Toledo). The lipid solutions were stored at −20 °C, while PBS was kept at room temperature.

### Preparation of the Experiment

Surface pressure–area (π–*A*) isotherms were measured using a fully automated mini-Langmuir trough (KSV NIMA, Finland; trough area = 24,300 mm^2^) as explained elsewhere.^20^ The trough was placed on an antivibration table inside a plexiglass box to prevent unwanted airflow and the introduction of dust particles onto the surface of the aqueous subphase. Prior to the experiment, the trough was cleaned using ethanol and chloroform: methanol (4:1, v/v) and rinsed twice with Milli-Q water. The trough was later filled with 130–140 mL of PBS 1X at pH 7.4. Subsequently, a Wilhelmy sensor and two barriers were installed in the trough. The Wilhelmy sensor is custom-made from Whatman paper No. 1, with a width of 10.30 mm. To control the subphase temperature, a thermostat (MGW Lauda Krüss) was used, operating through water circulation. To avoid temperature changes during the experiments, the subphase temperature was set at 20 ± 1 °C. All experiments were conducted isothermally.

### Isotherm Experiment

DPPC/POPC lipid mixtures were prepared in various mole fractions for POPC: 0, 0.25, 0.5, 0.75, and 1. Using a Hamilton syringe, 20 μL of the lipid solution was withdrawn and dropped evenly over the subphase between the two barriers. The solution was then left undisturbed for 15 min to allow evaporation of the solvent and stabilization of the system.

Observations were carried out at a constant temperature (20̊C), using the ″Constant Barrier Rate Compression″ mode. Each measurement was replicated a minimum of three times. For the isotherm experiments, the following parameters were employed according to our previous study:^21^ a compression/expansion rate of 10 mm/min, no residence time (barrier pause), and surface pressure targets for hysteresis set at 10 and 33 mN/m.

### Barrier Oscillation Experiment

The measurements (10 cycles) were conducted for all experiments. Three positions of surface pressure values, namely 2, 5, and 24 mN/m, were recorded. The experiment was based on both frequency sweep and amplitude sweep. For the sweeping frequency, seven frequencies were selected, i.e., 15, 25, 35, 50, 75, 100, and 125 mHz. For sweeping amplitude, 75 and 125 mHz were selected, while 0.5, 1, 3, 7, and 10% area amplitudes were chosen. All calculations were conducted automatically by the OscBarrier analysis program of the instrument KSV NIMA.

### AFM Experiments

Prior to observation, lipid films were transferred onto mica muscovite at at two distinct surface pressures—5 and 24 mN/m—maintained at 20 °C, according to Hruby et al.^22^ For lipid deposition, the mica was first affixed to a glass slide and then submerged into the subphase at an angle of approximately 30° relative to the air–water interface, with part of the glass slide aligned along the interface. Once the target surface pressure was reached, the mica was gradually withdrawn from the subphase to facilitate the monolayer deposition. Subsequently, AFM characterization was performed in contact mode (in air) using a Multimode apparatus (Veeco Bruker, MA, US) equipped with a 120 μm piezoelectric scanner. A triangular silicon nitride cantilever tip (DNP-S10) from Bruker was employed, with a 205 μm length and 40 μm width. The nominal spring constant of the cantilever was 0.12 N/m, with a tip radius ca. 10 nm. Meanwhile, the force applied was less than 1 nN. The resolution image was 512 × 512 pixels, and the scan rate was between 1.0 and 1.6 Hz. At least five replications per sample were measured for the reproducibility assessment. All images were processed by using Gwyddion 2.62.

## Results and Discussion

### Surface Pressure (π)–Area (*A*) Isotherms of Lipid Monolayers

We investigated the thermodynamic properties of DPPC-POPC monolayers spread on a Langmuir trough. The π–*A* isotherms, reflecting the thermodynamic properties of lipid monolayers, are shown in Figure 1a. All experiments were performed at 20 °C to observe a distinct coexistence phase transition of the DPPC monolayer, as noted in our previous study.^21^

**Figure 1:**
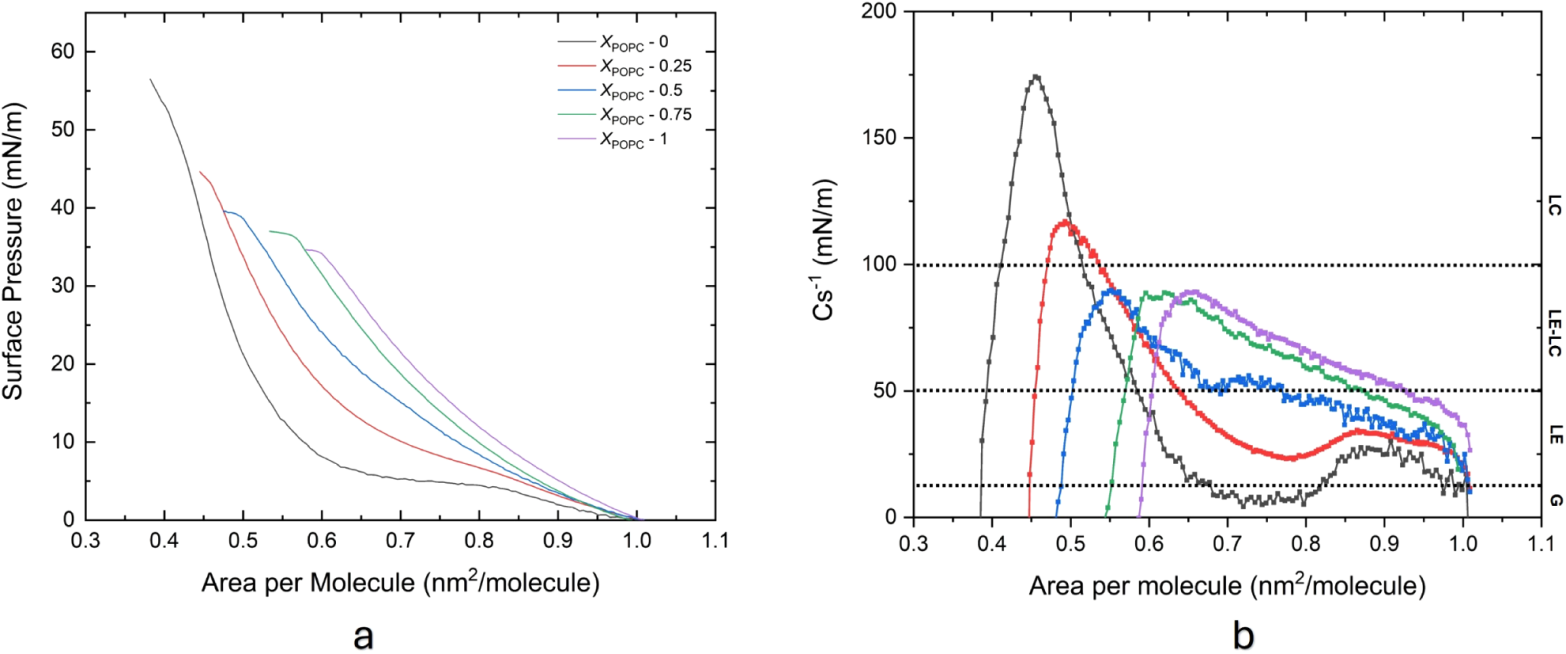
(a) Surface pressure (π)–area (*A*) isotherms for different DPPC/POPC mixtures and (b) compression modulus (Cs^–1^) versus surface pressure (π) for monolayers composed of different DPPC/POPC molar mixtures. The measurement was conducted at 20 °C on PBS subphase. The color of the curves represents the DPPC/POPC mixtures: black (*x*_POPC_ = 0), red (*x*_POPC_ = 0.25), blue (*x*_POPC_ = 0.5), green (*x*_POPC_ = 0.75), and purple (*x*_POPC_ = 1.0).

The coexistence/tilted phase transition will later serve as one of the distinguishing features when POPC is introduced into the DPPC monolayer. In its pure form, DPPC undergoes several phase states, moving from the liquid-expanded (LE) phase to a liquid-expanded–liquid-condensed (LE-LC) coexistence phase transition and then into a liquid-condensed (LC) phase. The DPPC isotherm is shown in black (Figure 1).

When POPC is introduced, the plateau of DPPC vanishes, except for the specific case of *x*_POPC_ = 0.25. The addition of POPC also results in an increase in the molecular area of the lipids. This behavior can be attributed to the presence of an unsaturated chain in POPC, which introduces a permanent kink in the acyl chain structure. This kink disrupts the tight molecular packing of the monolayer, which leads to increased fluidity compared to saturated lipids like DPPC.^23,24^ The increased chain disorder induced by the unsaturated bond facilitates a more homogeneous monolayer formation and enhances molecular mobility at the interface. Moreover, molecular dynamics simulations show that unsaturation reduces the tilt angle distributions and prevents the formation of highly ordered LC (liquid-condensed) domains. Consequently, it favors a more fluid and less tightly packed monolayer structure. This effect also lowers the LE-LC phase transition temperature of mixed DPPC/POPC monolayers and inhibits the development of gel-like domains at elevated temperatures.^24^

Furthermore, to achieve the same area per molecule, pure POPC and other lipid mixtures require higher compression compared to DPPC. For example, to reach an area per molecule of 0.7 nm^2^/molecule, DPPC only requires compression until a surface pressure of approximately 5 mN/m is achieved. However, for *x*_POPC_ = 0.25, it requires 10 mN/m, and this increases with higher POPC content, reaching up to pure POPC, which requires compression until the surface pressure reaches 20 mN/m.

To verify the phase behavior of the monolayer lipid mixtures, the compression modulus from the data of surface pressure (π)–area (*A*) isotherm curve was evaluated using the equation following:

$$Cs^{-1}=-A\left(\frac{\partial \pi }{\partial A}\right)$$

where *A* is the area per molecule at specific surface pressures (π). The compressive modulus is the ability of lipid monolayers to resist compression, as shown in Figures 1 and S1. A higher compression modulus value indicates greater resistance of the lipid film to deformation, while a lower value suggests the opposite.

According to Figure S1, DPPC (depicted by the black line) exhibits a distinct minimum in the compressibility modulus (Cs^1–^) around 5 mN/m. This marks the liquid-expanded (LE) to liquid-condensed (LC) phase transition. This observation is consistent with previous studies.^25,26^ While the π–A isotherm (Figure 1a) shows a plateau characteristic of this transition region, it is the modulus profile that more directly reflects the underlying mechanical change in the monolayer. Moreover, DPPC has the highest compression modulus compared with the other lipid mixtures, suggesting that it possesses greater rigidity and order (Figure 1b). In other words, the molecular arrangement of DPPC molecules is more densely packed.^27^

The introduction of POPC leads to several changes in the compression modulus-surface pressure curves: (1) it shifts the sharp minimum peak, and (2) it reduces the compressive modulus values. For instance, in the case of *x*_POPC_ = 0.25, the minimum peak shifts to around 8 mN/m, while for the rest of the lipid mixtures, the peak is less distinct or entirely absent. Similarly, for *x*_POPC_ = 0.25 the LC phase can still be observed, while it disappears for the rest of the compositions.

### Excess Gibbs Free Energy of Lipid Mixtures

To describe and explore the mixing behavior of mixed phospholipid monolayers, we quantified the excess Gibbs energy using the equation below:^28,29^

$$\Delta G_{\mathrm{exc}}=\int_{0}^{\pi }A_{\mathrm{exc}}\;d\pi =\int_{0}^{\pi }A_{\exp}-\left(A_{1}x_{1}+A_{2}x_{2}\right)d\pi$$

To observe the thermodynamic stability behavior of mixed lipid at specific surface pressure, the total Gibbs energy of mixing can be expressed using the following equation:

$$\Delta G_{\mathrm{mix}}=\Delta G_{\mathrm{exc}}+\Delta G_{\mathrm{ideal}}$$

$$\Delta G_{\mathrm{ideal}}=RT\;\left(x_{1}\ln x_{1}+x_{2}\ln x_{2}\right)$$

Where *A*_exp_ is the experimental mean molecular area of mixed lipid monolayer at discrete surface pressures, *A*_1_ and *A*_2_ are the mean molecular areas of pure DPPC and POPC, respectively, *x*_1_ and *x*_2_ are the mole fractions of the mixed lipid, *R* represents the ideal gas constant (8.314 J/mol·K), and *T* is the temperature of measurement in Kelvin (293 K).

Figure 2 shows that the excess Gibbs energy and total Gibbs energy are present. Excess Gibbs energy (Figure 2a) describes the deviation from ideal mixing. In other words, it reflects nonideal interactions in DPPC/POPC monolayers. For pure lipids, Δ*G*_exc_ is, by definition, equal to zero. This value indicates no deviation from ideality due to the absence of mixing. These zero points serve as a baseline reference. When Δ*G*_exc_ approaches zero at a certain composition in a lipid mixture, the interactions between different lipid molecules are comparable to those between identical molecules. If Δ*G*_exc_ is positive, the system deviates from ideal mixing due to unfavorable interactions. This means that DPPC and POPC prefer to interact with themselves rather than with each other. As a consequence, this suggests partial phase separation. On the other hand, if it is negative, mixing is more energetically favorable than expected for an ideal mixture, suggesting some preferential interactions between DPPC and POPC. In general, as the surface pressure increases, the distance between the lipid molecules decreases. This reduction in intermolecular distance enhances intermolecular interactions.^30–32^ Consequently, the miscibility of the lipid mixture improves, and the phase separation is reduced, as indicated by the decrease in Δ*G*_exc_. Notably, at mole fractions of POPC= 0.25 and 0.75, Δ*G*_exc_ is relatively low, suggesting that these mixtures exhibit stronger attractive interactions compared to a 0.5 mole fraction. This implies better miscibility in these nonequimolar mixtures. At equimolar composition (*x*_POPC_ = 0.5), the system may experience increased packing frustration due to the difference in the molecular structure of the alkyl chains between DPPC (saturated) and POPC (unsaturated). At intermediate ratios like 0.5, neither type dominates. This might lead to incompatibility in domain organization, resulting in less favorable mixing (reflected by a higher Δ*G*_exc_). In contrast, at low or high POPC fractions (0.25 or 0.75), the dominant lipid dictates the structural organization. The minority component may insert more easily into domains or remain in disordered regions, leading to lower excess free energy and better miscibility.

**Figure 2:**
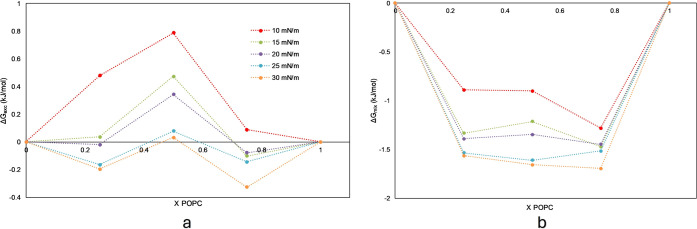
Excess Gibbs energy and Gibbs free energy of mixing for binary lipid DPPC/POPC mixture, concerning POPC composition at selected surface pressures. (a) excess Gibbs energy (Δ*G*_exc_), and (b) Gibbs free energy of mixing (Δ*G*_mix_).

Figure 2b presents the total Gibbs energy of mixing. This energy refers to the thermodynamic stability of the mixture. Analyzing Δ*G*_mix_ reveals that all lipid ratios yield negative values, indicating favorable states and confirming thermodynamic stability.

### Hysteresis Energy of a Binary Lipid Monolayer at Discrete Surface Pressures

Our study also explored the monolayer hysteresis phenomenon by performing compression and expansion cycles. Figures S2 and[Fig fig3] present the π–*A* isocycle curves and their calculated hysteresis energy, respectively. The calculation of the hysteresis energy was conducted using R-studio as follows:

$$\mathrm{Hysteresis}\;\mathrm{Energy}=\left|\int_{0}^{\pi }\mathrm{area}\;\mathrm{under}\;\mathrm{compression}\;\mathrm{curve}-\int_{\pi }^{0}\mathrm{area}\;\mathrm{under}\;\mathrm{expansion}\;\mathrm{curve}\right|$$

**Figure 3:**
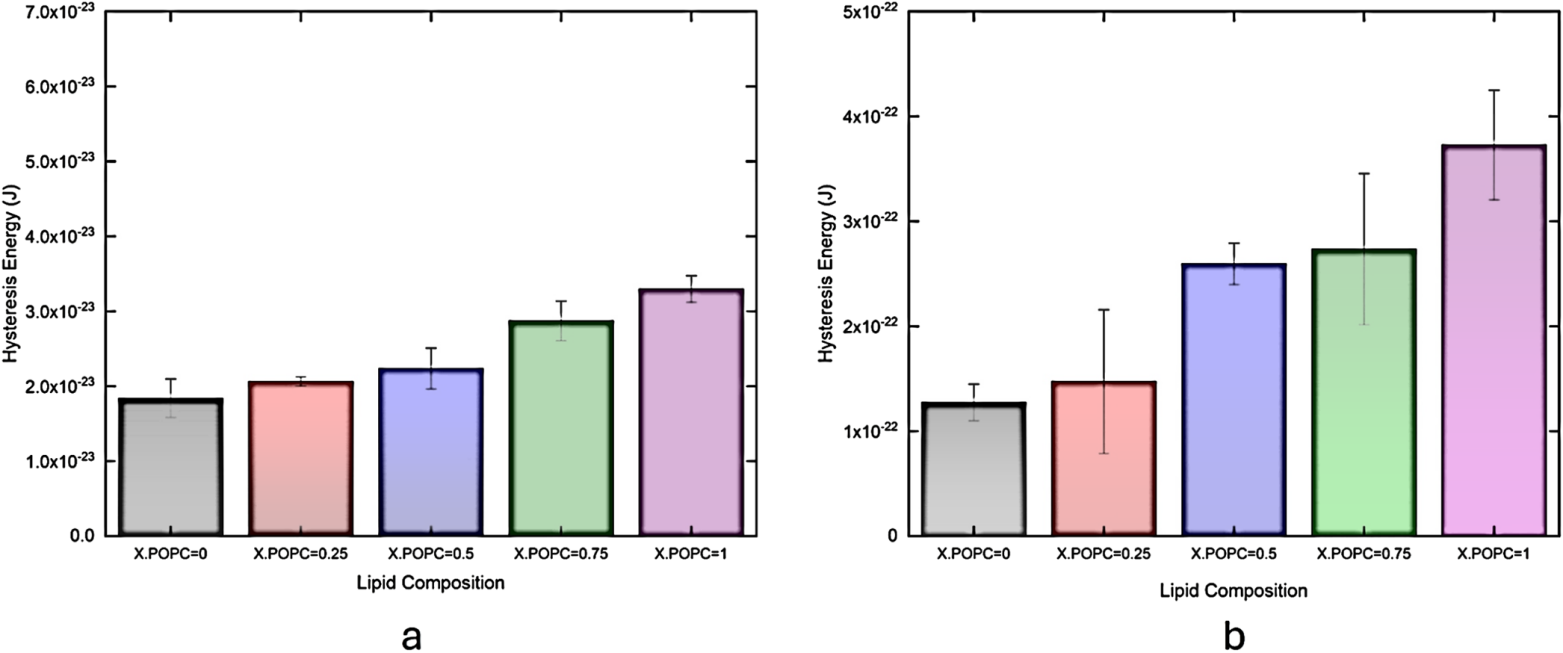
Hysteresis energy of binary lipid monolayers at discrete surface pressures of (a) 10 and (b) 33 mN/m. Note that each color corresponds to a concrete molar ratio of POPC in the mixture. The compression and expansion of π–*A* curves were measured at a rate of 10 mm/min.

The π–*A* isocycle curve includes a hysteresis loop, and on initial inspection, it bears a resemblance to that in Figure 1a. The disparities between the two figures lie in the target pressure and the decompression aspect. Numerous researchers have employed hysteresis modeling of lipids to study the resilience of the tear film lipid layer.^33–36^

Figure S2a,b highlight the film resilience of the lipid monolayers during compression and expansion. The hysteresis loop arises from alterations in the molecular packing of lipids. During compression, lipid molecules are packed more densely, and upon expansion, the molecules rearrange to restore the initial state. The area within the hysteresis loop represents the energy dissipated due to these molecular reorientations. This dissipation of energy indicates the resilience of the lipid film, which is dependent on lipid packing. All the phases observed in Figure 1a are also evident in the isocycle curve. We assessed hysteresis variations at specific surface pressures to understand how different molecular packing impacts are dissipated.

Figure S2 shows that lipid monolayers exhibit viscoelastic properties, as evidenced by the hysteresis loop. When the compression and expansion curves overlap (i.e., no deviation between both curves), the monolayer exhibits reversible behavior, typically seen in the liquid-condensed (LC) phase for saturated lipids, such as DPPC.^37^ However, when there is a deviation between the two curves, it indicates viscoelastic behavior, as observed along the coexistence region.

Figure 3a,b displays the hysteresis energy obtained from the hysteresis loop areas of the π–*A* isotherms shown in Figure S2a,b. We also calculated the hysteresis energy in J/mol to provide additional information on the dissipation energy of the lipid monolayer per mole, depicted in Figure S2c,d. Across all surface pressures, DPPC consistently shows the lowest hysteresis energy, while POPC presents the highest. At a surface pressure of 10 mN/m, the energy loss during the cyclic process lies within the range of 1.84 × 10^–23^ to 3.30 × 10^–23^ J. When the cyclic process is performed at a higher surface pressure of 33 mN/m, which is the surface pressure just before the POPC monolayer collapses, the hysteresis energy increases significantly, approximately 10-fold compared to that observed at low surface pressures. Notably, all hysteresis energies are lower than thermal energy (4.11 × 10^–21^J). This indicates that the driving force of molecular reorganization is low relative to the thermal energy, allowing molecules to rearrange toward equilibrium through expansion. However, the extent and rate of this rearrangement depend on lipid packing and interactions, which contribute to the observed hysteresis.

### Dilatational Rheology

Dilatational rheology is a crucial factor in evaluating the stability of thin films. By applying sinusoidal perturbation through barrier oscillation, the deformation of the lipid monolayer can be assessed. Sample sinusoidal wave curves are depicted in Figure S3.

Figure S3a illustrates the sinusoidal curves of the barrier oscillatory experiment, showing the relationship between the surface pressure, area per molecule, and time, while Figure S3b depicts the curves of the area per molecule versus time at 5 mN/m. According to Figure S3a,b, for the same area of deformation, the change in surface pressure is relatively small for DPPC at low surface pressure, followed by *x*_POPC_ = 0.25, *x*_POPC_ = 0.5, and *x*_POPC_ = 0.75, and POPC only.

To distinguish between the elastic and viscous contributions to the viscoelastic response, we separated the elastic and viscous components of the surface dilatational modulus, as illustrated in Figure 4. The magnitudes of the elastic and viscous moduli are of similar order. For example, at low surface pressure, the order of the elastic modulus from lowest to highest follows the trend from *x*_POPC_ = 0 to *x*_POPC_ = 1. A similar trend is observed for the viscous modulus. This suggests that pure POPC is more elastic than DPPC at low surface pressure, whereas the opposite behavior occurs at high surface pressure. However, the trends in the modulus values differ. Across all surface pressures studied, the viscous modulus generally increases with frequency (except for *x*_POPC_ = 0 at 2 mN/m), while the elastic modulus remains stable, with a slight decrease observed at 24 mN/m for *x*_POPC_ = 0, 0.25, and 1. This exhibits some minor frequency-dependent dissipative behavior in the viscous response.

**Figure 4:**
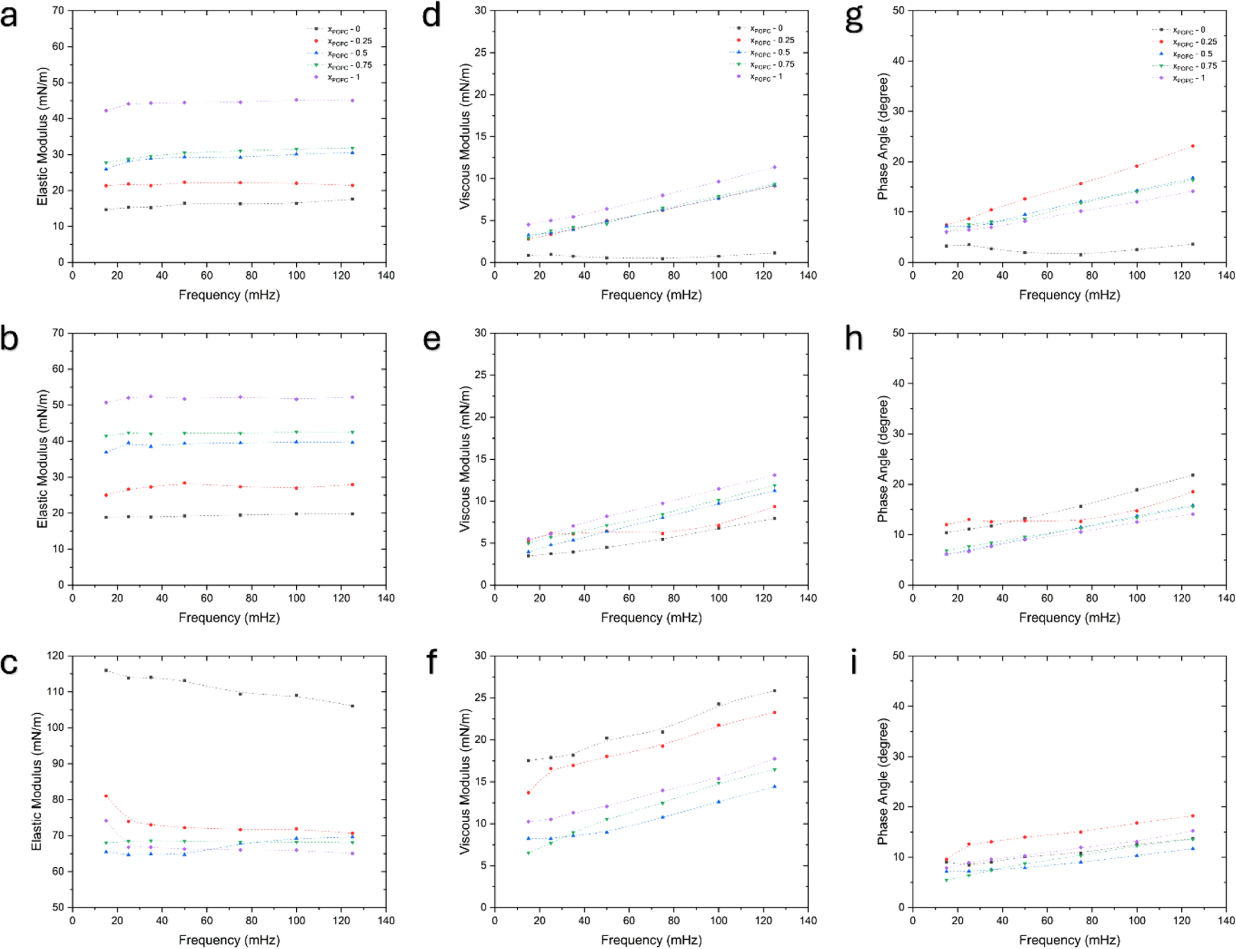
Viscoelastic properties of DPPC/POPC mixture monolayers during interfacial compression at various surface pressures. (a–c) Elastic modulus at surface pressures of (a) 2, (b) 5, and (c) 24 mN/m. (d–f) Viscous modulus at surface pressures of (d) 2, (e) 5, and (f) 24 mN/m. (g–i) Phase angle at surface pressures of (g) 2, (h) 5, and (i) 24 mN/m. The compression and expansion cycles were performed in a range of frequencies from 15 to 125 mHz. The codes for symbols are as follows: black square (*x*_POPC_ – 0), red circle (*x*_POPC_ – 0.25), blue triangle (*x*_POPC_ – 0.5), inverted green triangle (*x*_POPC_ – 0.75), and purple rhombus (*x*_POPC_ – 1).

The results also show that the elastic modulus is consistently greater than the viscous modulus across all surface pressures and frequencies. Even the highest viscous modulus does not exceed the lowest elastic modulus across the selected surface pressures. This is typical behavior for lipid films and surfactants, where the elastic modulus is generally higher than the viscous modulus.^38,39^ These findings suggest that the monolayers exhibit predominantly elastic behavior.

Additionally, the phase angle (δ) was calculated to evaluate the ratio of viscous to elastic responses. In general, the phase angles were low, ranging from 1° to 22° at 2 mN/m and from 5° to 20° at 5 mN/m, showing similar values at 24 mN/m. The small phase angles reinforce the idea that the monolayers behave largely elastically under the studied conditions. The trend of the phase angle mirrors that of the viscous modulus, increasing with frequency, although the order of the values is slightly different. For instance, at 2 mN/m, the highest value of viscous modulus is *x*_POPC_ = 1, whereas the highest angle at a similar surface pressure is *x*_POPC_ = 0.25. Similarly, although *x*_POPC_ = 1 exhibits the highest viscous modulus at 5 mN/m, it shows the lowest phase angle at the same pressure.

The total modulus of dilatational viscoelasticity of the lipid mixtures was then calculated, as shown in Figure 5. To determine the effect of amplitude, we also measured the surface dilatational modulus under different amplitudes (Figure 6). Our findings for the pure DPPC dilatational modulus align with previous studies,^40–43^ which conducted experiments using the oscillating drop and Langmuir techniques.

**Figure 5:**
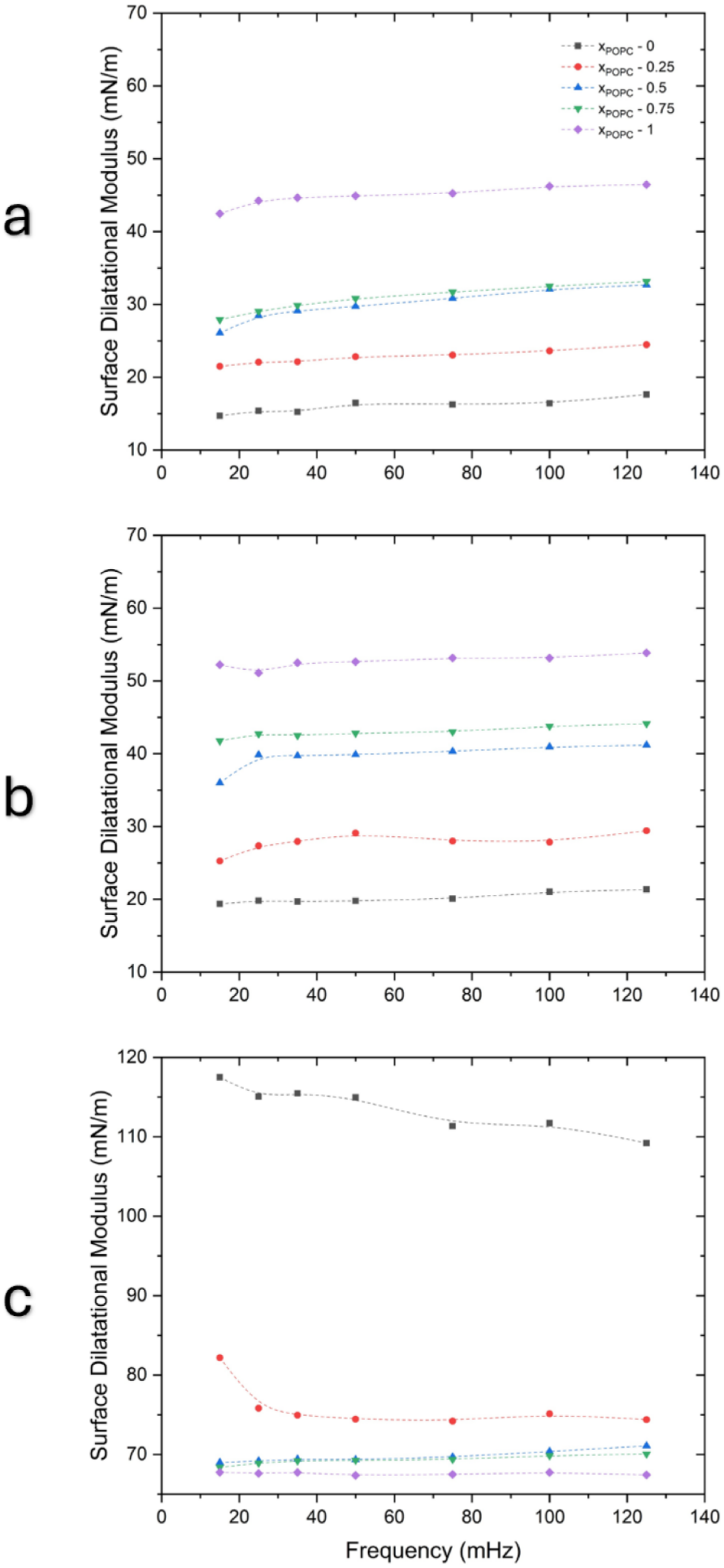
Surface dilatational modulus of DPPC/POPC mixture monolayers during interface compression at surface pressures of (a) 2, (b) 5, and (c) 24 mN/m. The compression and expansion cycles were performed in a range of frequencies from 15 to 125 mHz. The codes of symbols are as follows: black square (*x*_POPC_ – 0), red circle (*x*_POPC_ – 0.25), blue triangle (*x*_POPC_ – 0.5), inverted green triangle (*x*_POPC_ – 0.75), and purple rhombus (*x*_POPC_ – 1).

**Figure 6:**
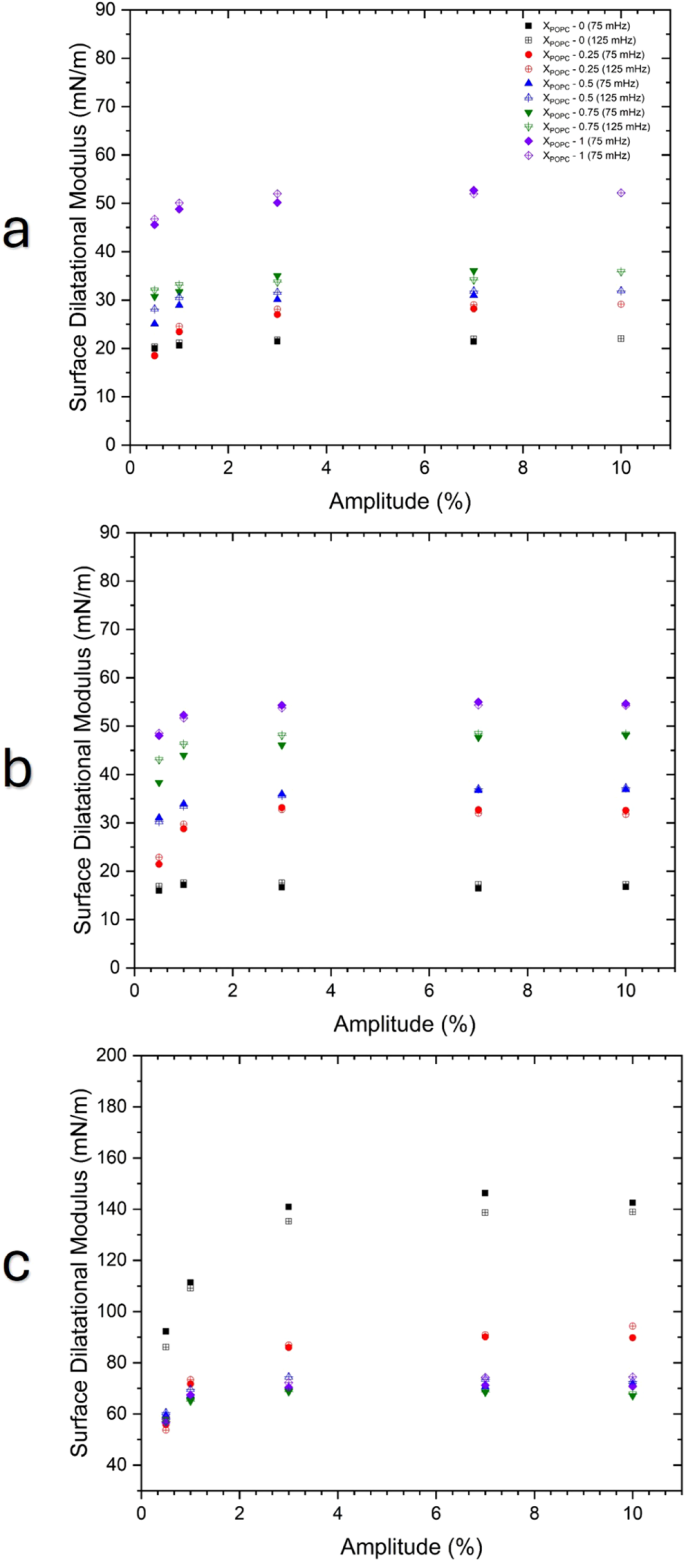
Dilatational rheological modulus of DPPC/POPC mixture monolayers corresponding to lipid deformation with amplitudes ranging from 0.5 to 10% at surface pressure of (a) 2 mN/m, (b) 5 mN/m, and (c) 24 mN/m. Figure a only provides the amplitude up to 7.5% for a frequency of 125 mHZ due to the limit area of Langmuir trough. Black color symbols: *x*_POPC_ – 0, red color symbols: *x*_POPC_ – 0.25, blue color symbols: *x*_POPC_ – 0.5, green color symbols: *x*_POPC_ – 0.75, and purple color symbols: *x*_POPC_ – 1. Filled symbols are the measurement at 75 mHz while opened patterned symbols are the measurement at 125 mHz. The codes for symbols: ■ (*x*_POPC_ – 0), ● (*x*_POPC_ – 0.25), ▲ (*x*_POPC_ – 0.5), ▼ (*x*_POPC_ – 0.75), and ⧫ (*x*_POPC_ – 1).

As shown in Figure 5a,b, at low surface pressure (2 and 5 mN/m), the trend of the dilatational modulus is similar, ranging between 15 and 60 mN/m. Starting from the lowest values, DPPC exhibits the smallest modulus, followed by *x*_POPC_ = 0.25, *x*_POPC_ = 0.5, *x*_POPC_ = 0.75, and finally pure POPC. For all lipid mixtures, the dilatational moduli remain almost constant over the selected frequency. In such material, energy is stored during deformation and is almost fully recovered without significant dissipation. However, at lower frequencies, such as between 0.001 and 0.01 Hz, as reported by Arriaga et al., the surface pressure in the LE-LC region shows viscoelastic properties.^44^ At low frequencies, the monolayer molecules have more time to reorganize.

A different pattern appears at a higher surface pressure (Figure 5c), i.e., 24 mN/m, although this behavior underlines the lipid mixtures. The moduli values range from POPC to DPPC, with POPC having the lowest values and DPPC the highest. Again, the addition of POPC molecules makes the monolayer less compressible and less elastic. Adding more POPC to DPPC decreases the overall elasticity. With a larger proportion of POPC, the modulus is relatively low compared to DPPC-rich mixtures.

Furthermore, to understand the mechanical properties of the lipid monolayers, we compare the compression modulus (Figure 1b) with the surface dilatational modulus at three fixed surface pressures (2, 5, and 24 mN/m). Although these parameters reflect different types of deformation and physical meanings, they are both related under certain conditions—especially during changes in packing density. Across all compositions, a consistent trend is observed: monolayers with a higher compression modulus at selected surface pressure also exhibit higher surface dilatational modulus values. For instance, at high surface pressures, pure DPPC (x_POPC_ = 0) exhibits the highest values for both the compression modulus and the surface dilatational modulus, while pure POPC (*x*_POPC_ = 1) shows the lowest. In other words, both moduli increase for DPPC in the condensed phase because the film becomes more rigid and resists both static and dynamic deformations. Conversely, at low surface pressures, pure POPC displays the highest compression and surface dilatational moduli, whereas pure DPPC shows the lowest, since it is less elastic in this regime. Interestingly, the surface dilatational modulus values are generally similar to the compression modulus values, except for DPPC at 5 mN/m.

### Amplitude Sweep

Amplitude sweeping is less commonly employed in interfacial rheology than in bulk rheology. This is because thin films are typically brittle and more susceptible to deformation than bulk materials, especially at high amplitudes.^45^ In Langmuir experiments, when sweeping the amplitude, it is crucial to maintain the integrity of the monolayer and avoid artifacts such as the formation of waves or ripples in the subphase. To prevent such issues, the amplitude of the perturbation should generally be kept within a small range, relative to the overall area of the monolayer. Therefore, we conducted the amplitude sweeping experiment up to 10%, as depicted in Figure 6.

As can be seen, Figure 6 shows the dilatational moduli as a function of amplitude, ranging from 0.5% up to 10% at three different surface pressures, i.e., 2, 5, and 24 mN/m, for frequency oscillations of 75 and 125 mHz. For all lipid mixtures, the trend of the moduli for 75 mHz and 125 mHz is similar. It is worth noting that changes in amplitude can significantly affect the elasticity or viscoelasticity of monolayers.

At low amplitude, the monolayer is subjected to relatively minor perturbations. Increasing the amplitude introduces more significant perturbations, which can lead to larger deformations and potential changes in the monolayer’s structural integrity. This may result in increased observed moduli, as the monolayer may become stiffer under larger oscillations or because the larger amplitude might be affecting the molecular packing. More precisely , it seems that the threshold for hardening the membrane occurs at an amplitude of 2.5%, based on an experiment conducted. Later on, the increase in amplitude does not confer any significant modulus shift. This phenomenon follows type II LAOS (Large Amplitude Oscillatory Shear)—strain hardening. Strain hardening, also known as strain stiffening, is thought to arise from the development of intricate microstructures within the material. This process involves the formation of nonlinear elastic elements that create a reinforced network structure.^46^ This phenomenon is also observed in F-actin, fibrin, and collagen systems.^47^

We also evaluated how the monolayers respond to oscillatory stress via Lissajous plots. Essentially, Lissajous plots are similar to hysteresis experiments, with differences in the number of barrier oscillations and the size of the hysteresis area. As shown in Figures 7 and S4, Lissajous plots offer a clear way to visualize nonlinear behavior during cyclic deformation. Kempen et al. showed how Lissajous plots can be used to define the nonlinear surface dilatational rheology of oligofructose fatty acid esters.^45^ Groot et al. demonstrated that Lissajous plots describe the nonlinear interfacial properties of protein-complex mixture in large-amplitude oscillatory dilatation,^48^ while Gimenez-Ribes et al. analyzed the interfacial rheology and relaxation of the saponin Escin using this method.^49^

**Figure 7:**
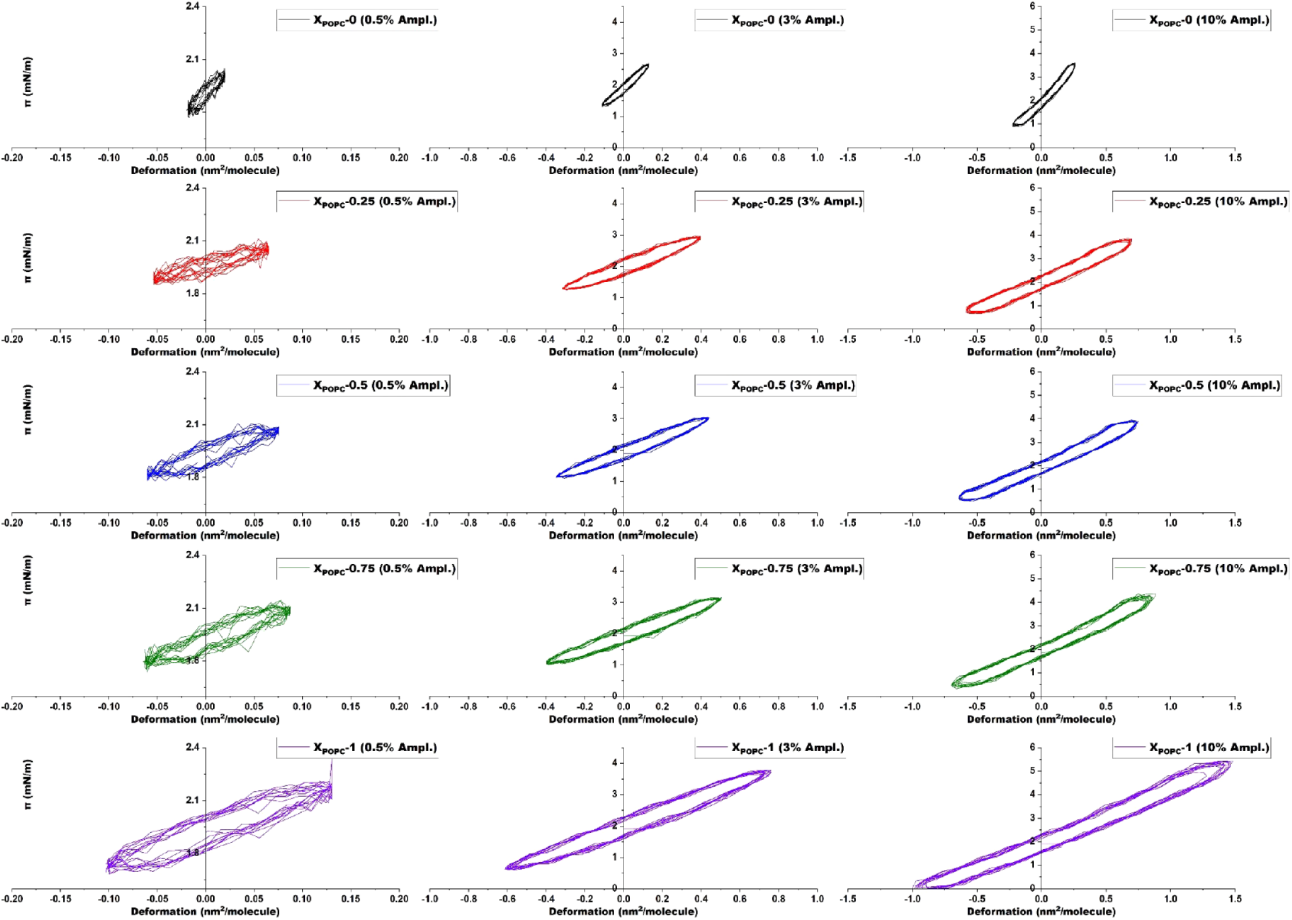
Lissajous plots as a function of amplitude sweep for DPPC/POPC at 20 °C at a surface pressure of 2 mN/m. Black color curves: *x*_POPC_ – 0, red color curves: *x*_POPC_ – 0.25, blue color curves: *x*_POPC_ – 0.5, green color curves: *x*_POPC_ – 0.75, and purple color curves: *x*_POPC_ – 1.

The shape of the plots mainly depends on the lipid mixture and the given amplitude. Figure 7 shows the Lissajous plots of lipid mixtures at 2 mN/m with an amplitude sweep of 0.5%, 3%, and 10%. At this surface pressure, aligned with the findings in Figure S4, the monolayer is predominantly elastic. In a purely elastic material, the plot will show a straight line and be symmetric because stress and strain are in phase. Regarding linearity, at small oscillation amplitudes, including those in this study, may exhibit linear behavior. For example, DPPC shows deformation between −0.02 and 0.02, and its Lissajous plot produces an elliptical curve, indicating that the material responds predictably to stress with minimal energy dissipation.

However, when the amplitude increases, the deformation is not perfectly linear. For instance, when DPPC is swept with a 10% amplitude, it deforms between −0.2 and 0.3. The plot is no longer a perfect ellipse and becomes more distorted, indicating that the material is transitioning to nonlinear behavior. At higher amplitudes, the monolayer experiences larger deformations, which cause reorganization of the molecules within the monolayer. Increasing the fluidity also affects the linearity. For example, the deformation of x_POPC_ = 0.5 lies between −0.06 and 0.075, and the deformation increases with the increase in POPC ratio. Increased fluidity indicates a lack of interfacial viscosity. Consequently, the monolayer’s integrity at the interface is disturbed, resulting in larger deformations under the same applied stress.^50^ This also shows that monolayers dissipate greater energy, consistent with the findings in Figure S2. Furthermore, the nonlinearity can be seen clearly at a surface pressure of 24 mN/m, particularly for high amplitudes. The ellipse shape is more distorted for pure DPPC.

### Microstructures

To evaluate the addition of POPC to the DPPC monolayer and to clarify the immiscibility of both lipids, we imaged the micro- and nanostructures of lipid monolayers using AFM, as depicted in Figure 8. To better visualize the surface morphology and domain structures, representative three-dimensional (3D) AFM images are provided in Figure S5. Figure 8a–e shows the structure of the lipid monolayers at 5 mN/m, while Figure 8f–j shows the structure of lipid monolayers at 24 mN/m. In Figure 8, the macrodomains of lipid monolayers are evident for all lipid mixtures except POPC. At the specific surface pressure at which DPPC exhibits the LC-LE phase transition, it can be deduced that the macrodomain corresponds to the LC phase of DPPC (see the DPPC image), while the surrounding area represents the LE phase. The topography of the monolayer does not definitively confirm that the uncovered areas (without domains) correspond to the mica surface; they could potentially represent the first lipid monolayer as the LE phase.^51^

**Figure 8:**
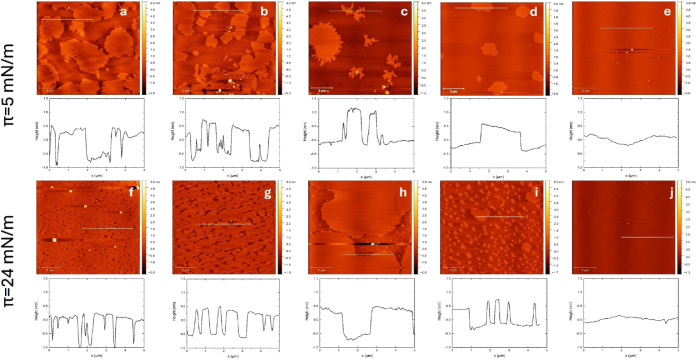
AFM images of lipid monolayers at specific surface pressures of (top) 5 and (bottom) 24 mN/m, and their profiles (along the white line) for different molar POPC fractions. (a, f) *x*_POPC_ = 0, (b, g) *x*_POPC_ = 0.25, (c, h) *x*_POPC_ = 0.5, (d, i) *x*_POPC_ = 0.75, and (e, j) *x*_POPC_ = 1.

For the DPPC monolayer, the average maximum domain height is 1.6 ± 0.1 nm relative to the surrounding LE phase (i.e., areas without domains), as evaluated using Gwyddion software (version 2.62). It is important to note that the height obtained from AFM imaging represents the relative difference in elevation (thickness) between LC-phase domains and the adjacent LE phase rather than the absolute monolayer thickness. In binary mixtures, LC-phase domains are still visible for all compositions except pure POPC. However, the measured domain height decreases with an increasing POPC mole fraction. This indicates an increasing disruption of the ordered LC phase. The average maximum domain heights for *x*_POPC_ = 0.25, 0.5, and 0.75 were found to be 1.14 ± 0.13 nm, 1.13 ± 0.20 nm, and 0.77 ± 0.21 nm, respectively. This decline in thickness supports the notion that up to *x*_POPC_ = 0.5, the film retains DPPC-rich domains of relatively constant structure, but at higher compositions (e.g., *x*_POPC_ = 0.75), the continuity of these domains is compromised. It is worth mentioning that there is a compositional threshold beyond which POPC significantly alters the monolayer morphology.

Figure 8f shows that when DPPC reaches the LC phase, all of the macrodomains of DPPC aggregate to form abundant large macrodomains, and small macrodomains are no longer formed). Some small holes (around 150 nm) are still visible, while the profile image shows a lipid thickness of about 1 nm. This phenomenon is also observed in other lipid mixtures. The domains become visible at a surface pressure of 5 mN/m. As the pressure increases, the lipid domains move closer to each other, forming larger domains, as observed for POPC molar fractions of 0.5 and 0.75.

As the amount of POPC added increases, the liquid-expanded phase of POPC becomes more dominant (Figure 8g–j). Eventually, the macrodomains become more pronounced, and even some microdomains appear scattered, as can be seen at mole fractions of *x*_POPC_ = 0.5 and 0.75. On the other hand, both macrodomains and microdomains are no longer visible when the monolayer consists only of pure POPC.

## Conclusions

In our study, we utilized the Langmuir technique to assess the thermodynamic properties and interfacial dilatational rheology of mixed DPPC/POPC lipid systems. Our results reveal that lipid–lipid interactions significantly impact both the viscoelasticity and the stability of the monolayer. The presence of POPC in the lipid mixture altered the phase states, leading to an increase in the hysteresis energy, which reflects changes in the molecular packing and viscoelastic behavior. We also highlighted the importance of surface rheology in the deformation of the lipid monolayer. The surface rheology remains constant at selected frequencies and low amplitudes, but increasing amplitude triggers greater deformations. The phase angles present low values, which indicate an elastic behavior. Lissajous plots proved to be an effective tool in characterizing the nonlinear behavior of the lipid monolayers, particularly at higher amplitudes. We also present observations of the monolayer microstructure, showing that the macrodomain of DPPC is scattered due to the addition of POPC, while the thickness of the domains is reduced. Notably, a strong correlation was observed between the surface dilatational modulus and compression modulus, suggesting that both parameters reflect changes in monolayer rigidity under different packing densities. Overall, this study enhances our understanding of the complex interplay of mixed lipids in monolayers and offers crucial information on their behavior under varying surface pressures and deformation.

## Supplementary Material



## Notes

The curves of compression modulus versus surface pressure of DPPC/POPC; isocycle curves and hysteresis energies; sinusoidal waves from barrier oscillation experiment; Lissajous plots at a surface pressure of 24 mN/m; and 3D AFM images of lipid films (PDF)
