# Supplementary material for: Surface Rheological Properties and Microstructures of DPPC/POPC Monolayers
Source: Langmuir. 2025 Jun 20;41(25):16128–38. doi: 10.1021/acs.langmuir.5c01269 (PMC12224327; doi:10.1021/acs.langmuir.5c01269)
Supplement: Supplementary file 1 [file la5c01269_si_001.pdf]

# Supporting Information

# Surface Rheological Properties and Microstructures of DPPC/POPC Monolayers

*Wisnu Arfian Anditya Sudjarwo*[1] * *and Jose Luis Toca-Herrera*[1] *

[1]Institut für Biophysik, Universität für Bodenkultur Wien (BOKU), 1190 Vienna, Austria

Corresponding authors: wisnu.sudjarwo@boku.ac.at, jose.toca-herrera@boku.ac.at

Number of pages : 6

Number of figures : 5

Number of schemes : 0

Number of tables : 0

Table of contents :

**Figure S1.** The curves of compression modulus versus surface pressure of lipid mixtures

**Figure S2.** The isocycle curves and hysteresis energies of lipid monolayers

**Figure S3.** The sinusoidal waves from barrier oscillation experiment at 5 mN/m

**Figure S4.** Lissajous plots of lipid mixtures at 24 mN/m

**Figure S5.** 3D AFM images of lipid mixtures

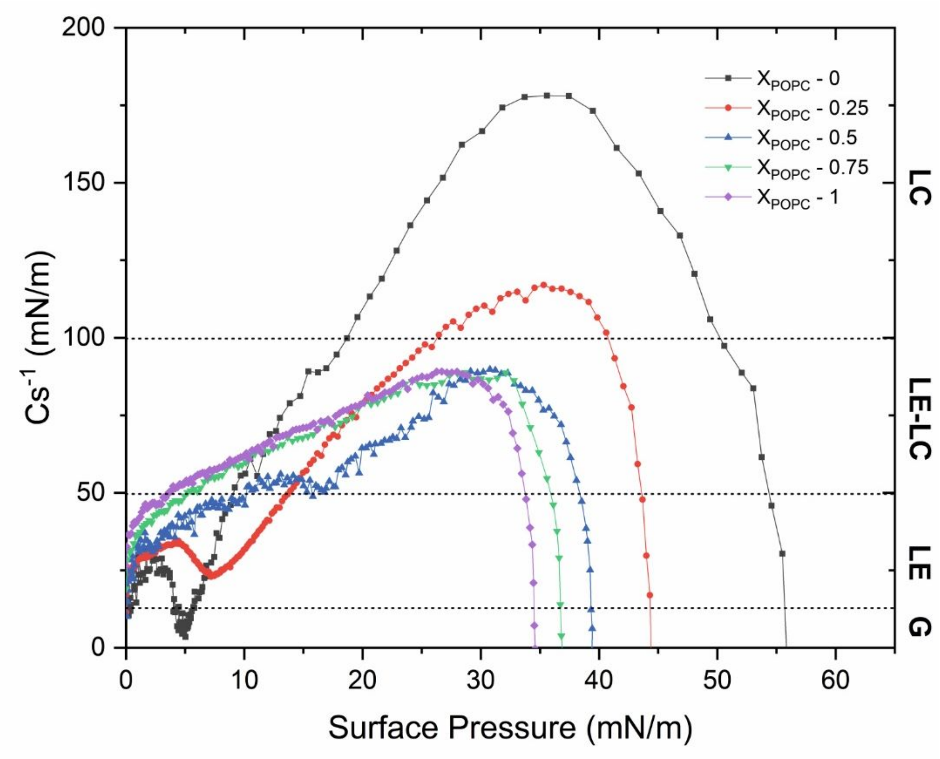


**Figure S1**. The curve of compression modulus ($Cs^{-1}$) versus surface pressure (π) for monolayers composed of different DPPC/POPC molar mixtures. The measurement was conducted at 20˚C on PBS subphase.

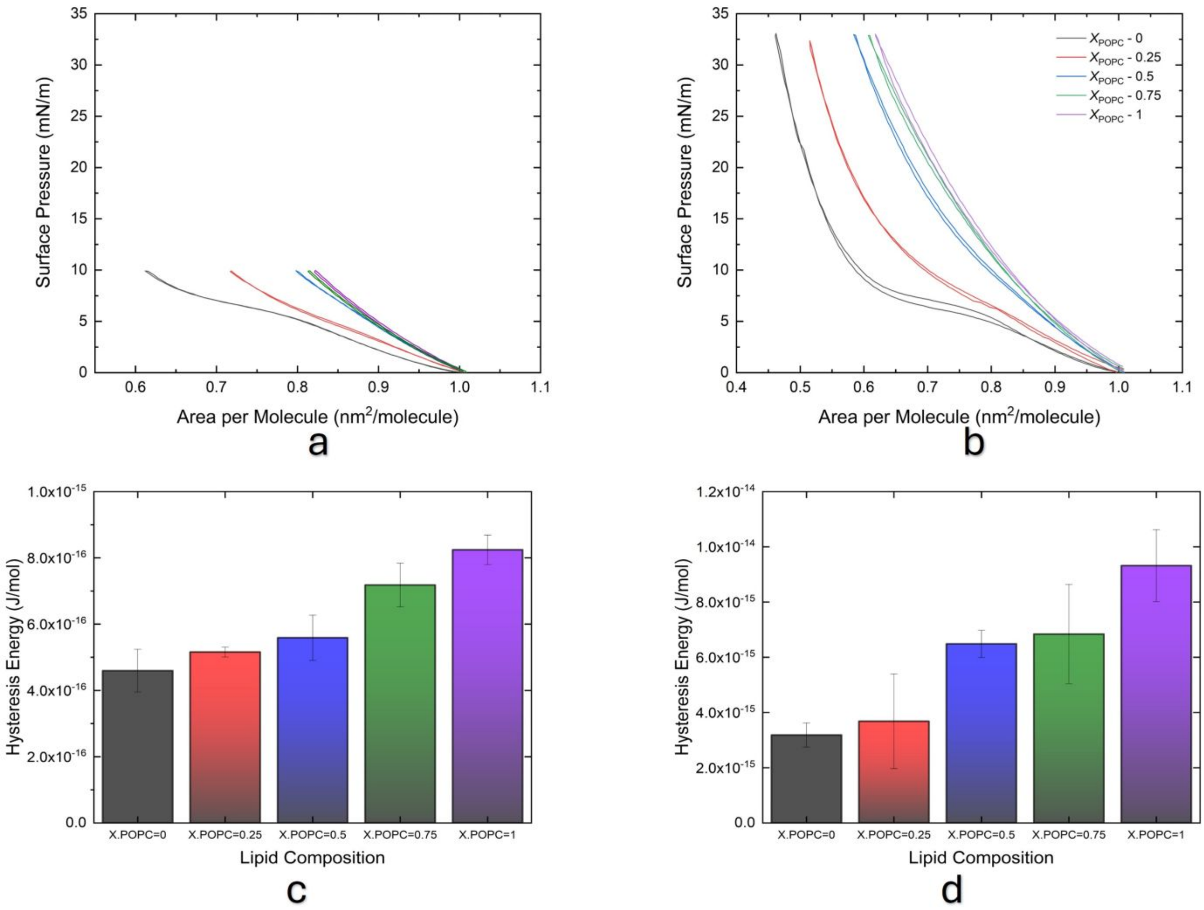


**Figure S2.** The π-A isotherm isocycle curve of lipid monolayer at discrete surface pressure of a) 10 mN/m and b) 33 mN/m, and hysteresis energy of lipid monolayer at discrete surface pressure of c) 10 mN/m and d) 33 mN/m. Note that every color corresponds to a concrete molar ratio of POPC in the mixture. The compression and expansion of π-A curves were measured at a rate of 10 mm/min.

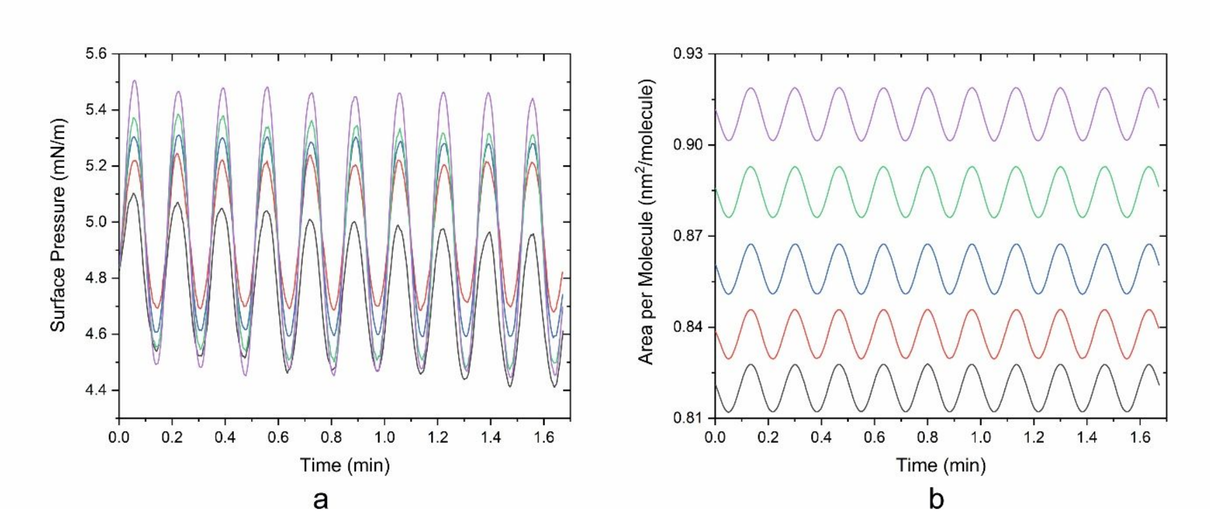


**Figure S3.** The examples of sinusoidal waves from barrier oscillation experiment at 5 mN/m between **a)** surface pressure vs. time, **b)** area of molecule vs. time. Black curve: $X_{POPC}$ – 0, red curve: $X_{POPC}$ – 0.25, blue curve: $X_{POPC}$ – 0.5, green curve: $X_{POPC}$ – 0.75, and purple curve: $X_{POPC}$ – 1

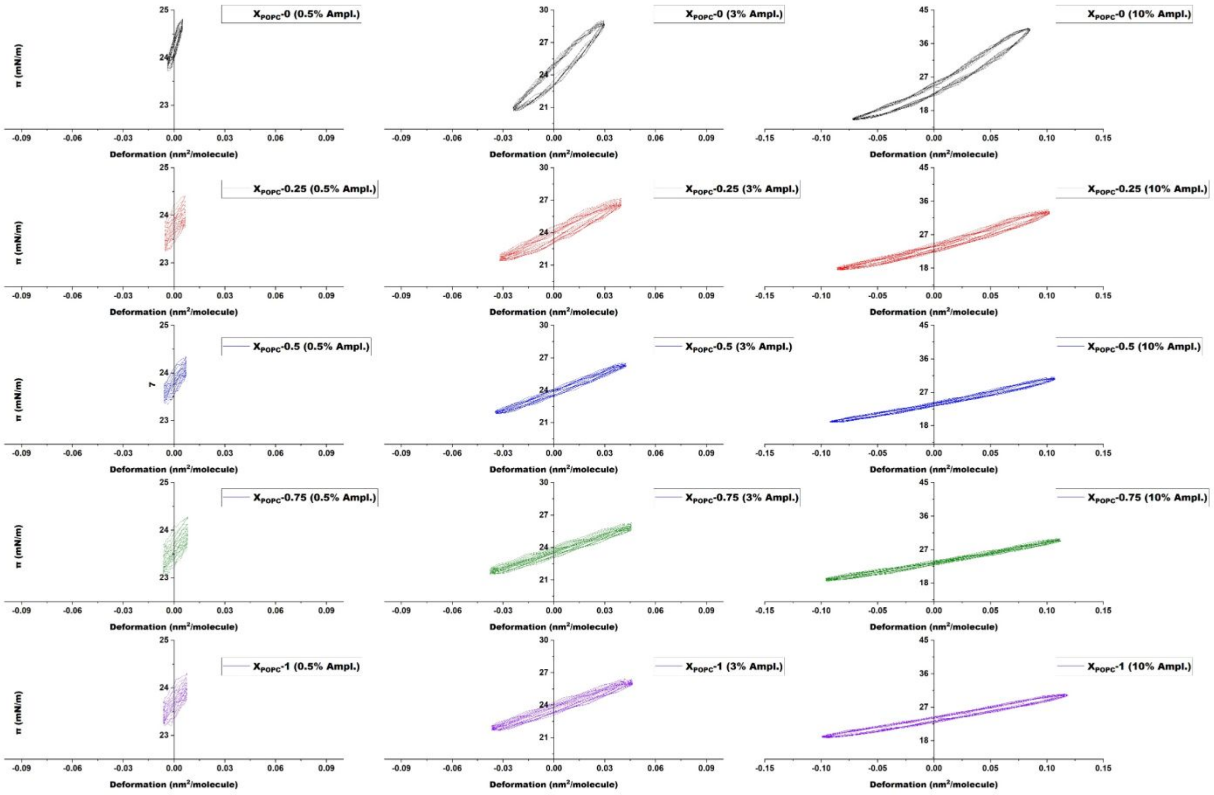


**Figure S4.** Lissajous plots as a function of amplitude sweep for DPPC/POPC at 20$^{o}$C at surface pressure of 24 mN/m. Black curve: $x_{POPC}$ – 0, red curve: $x_{POPC}$ – 0.25, blue curve: $x_{POPC}$ – 0.5, green curve: $x_{POPC}$ – 0.75, and purple curve: $x_{POPC}$ – 1

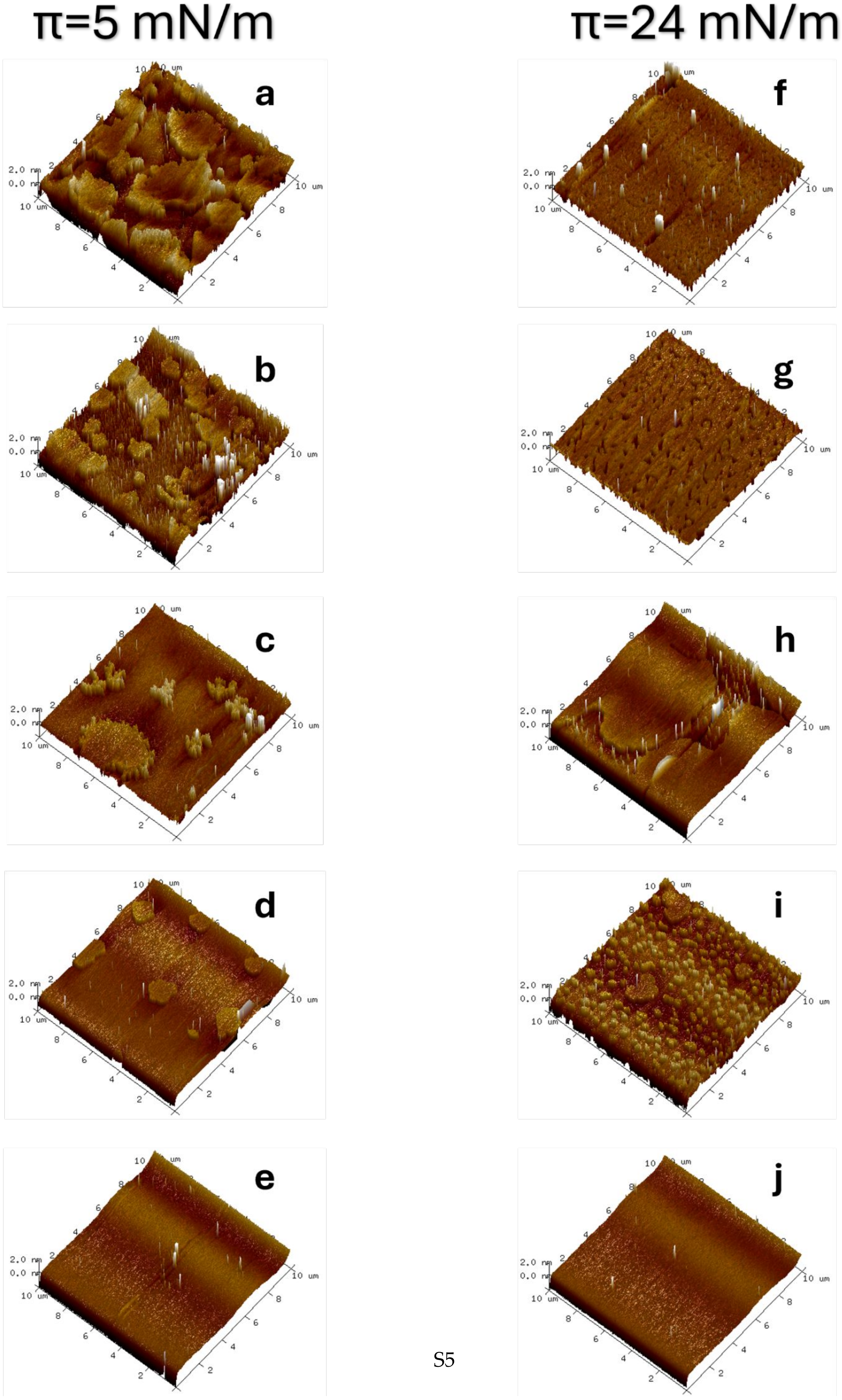
π=5 mN/m
π=24 mN/m
a
b
c
d
e
f
g
h
i
j

**Figure S5**. Three-dimensional AFM images of lipid monolayers at specific surface pressures of (top) 5 mN/m and (bottom) 24 mN/m, and their profiles for different molar POPC fractions. a,f) $x_{POPC}$=0, b,g) $x_{POPC}$=0.25, c,h) $x_{POPC}$=0.5, d,i) $x_{POPC}$=0.75, and e,j) $x_{POPC}$=1.
